# Supplementary figures and images for: ciRS-7 exonic sequence is embedded in a long non-coding RNA locus
Source: PLoS Genet. 2017 Dec 13;13(12):e1007114. doi: 10.1371/journal.pgen.1007114 (PMC5745005; doi:10.1371/journal.pgen.1007114)

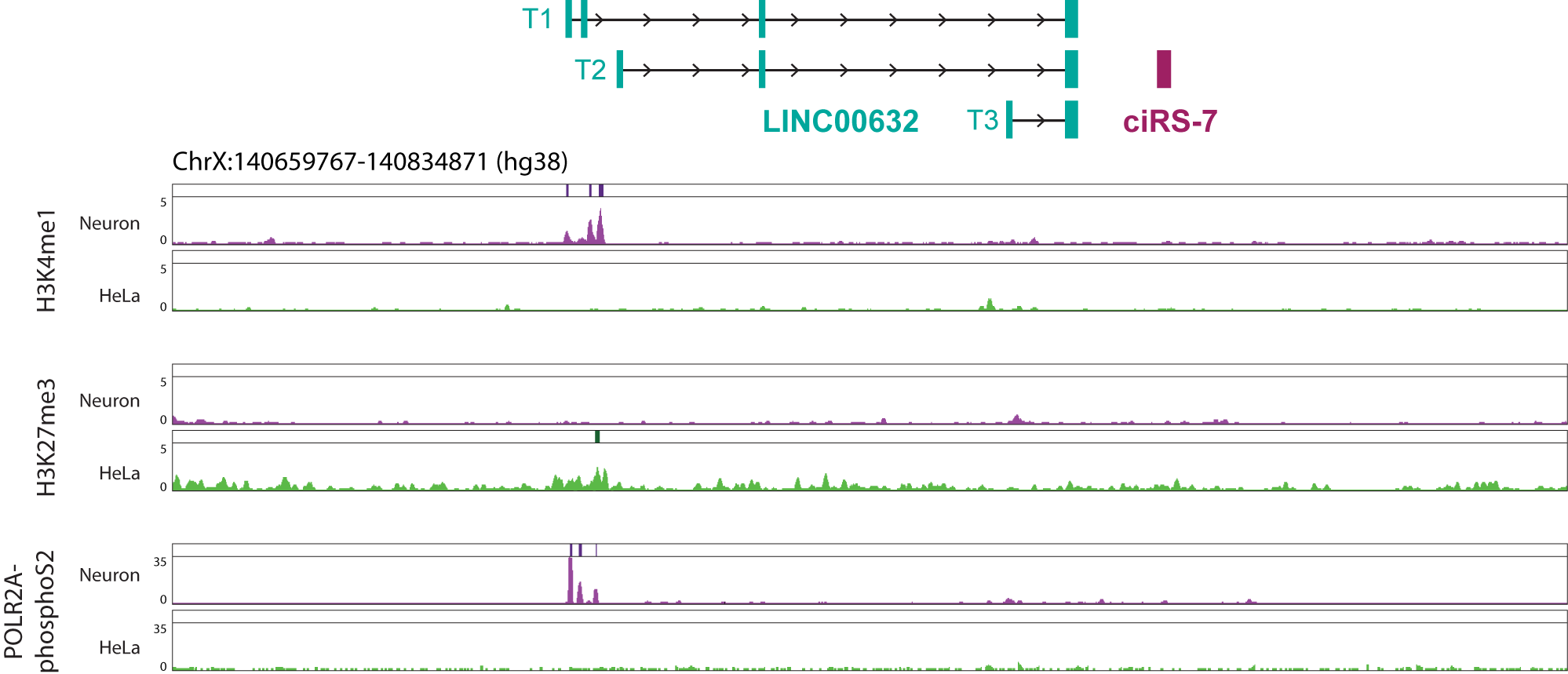

Supplement: S1 Fig — (TIF) [file pgen.1007114.s001.tif]

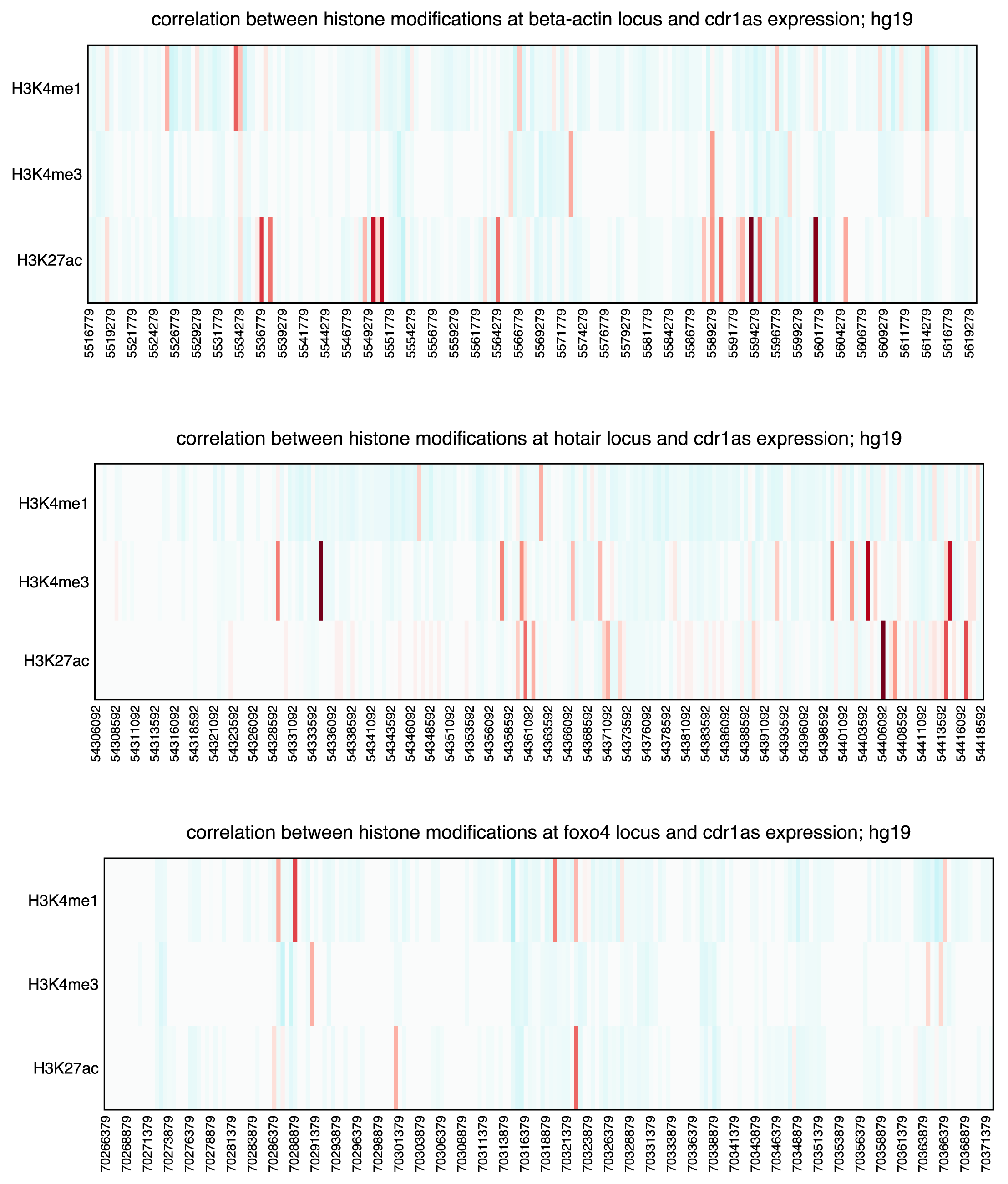

Supplement: S2 Fig — Coordinates reported are from the hg19 genome build. (TIF) [file pgen.1007114.s002.tif]

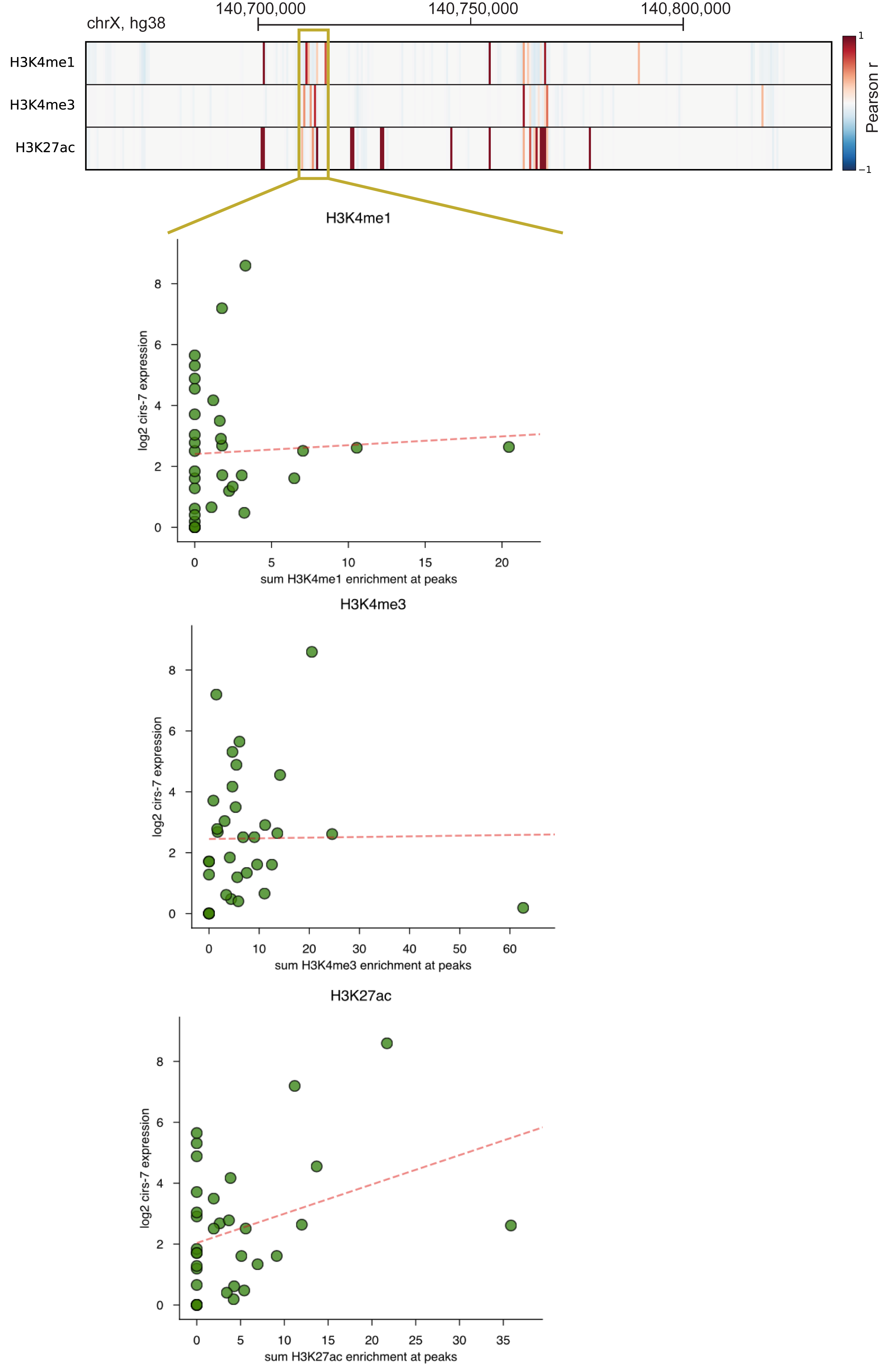

Supplement: S3 Fig — (TIF) [file pgen.1007114.s003.tif]

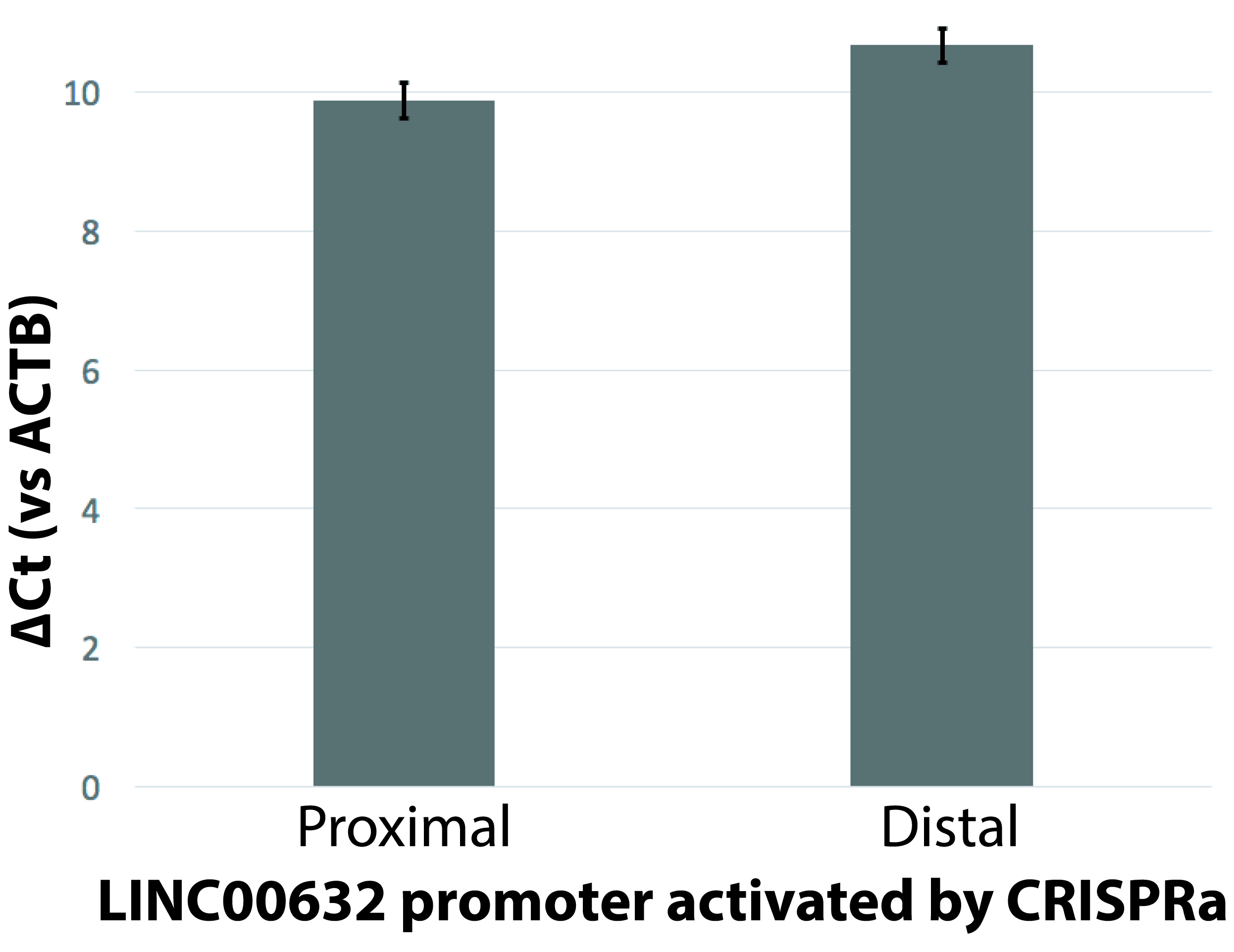

Supplement: S4 Fig — qPCR measurement of ciRS-7 expression relative to actin. Error bars represent the standard deviation of biological replicates. (TIF) [file pgen.1007114.s004.tif]

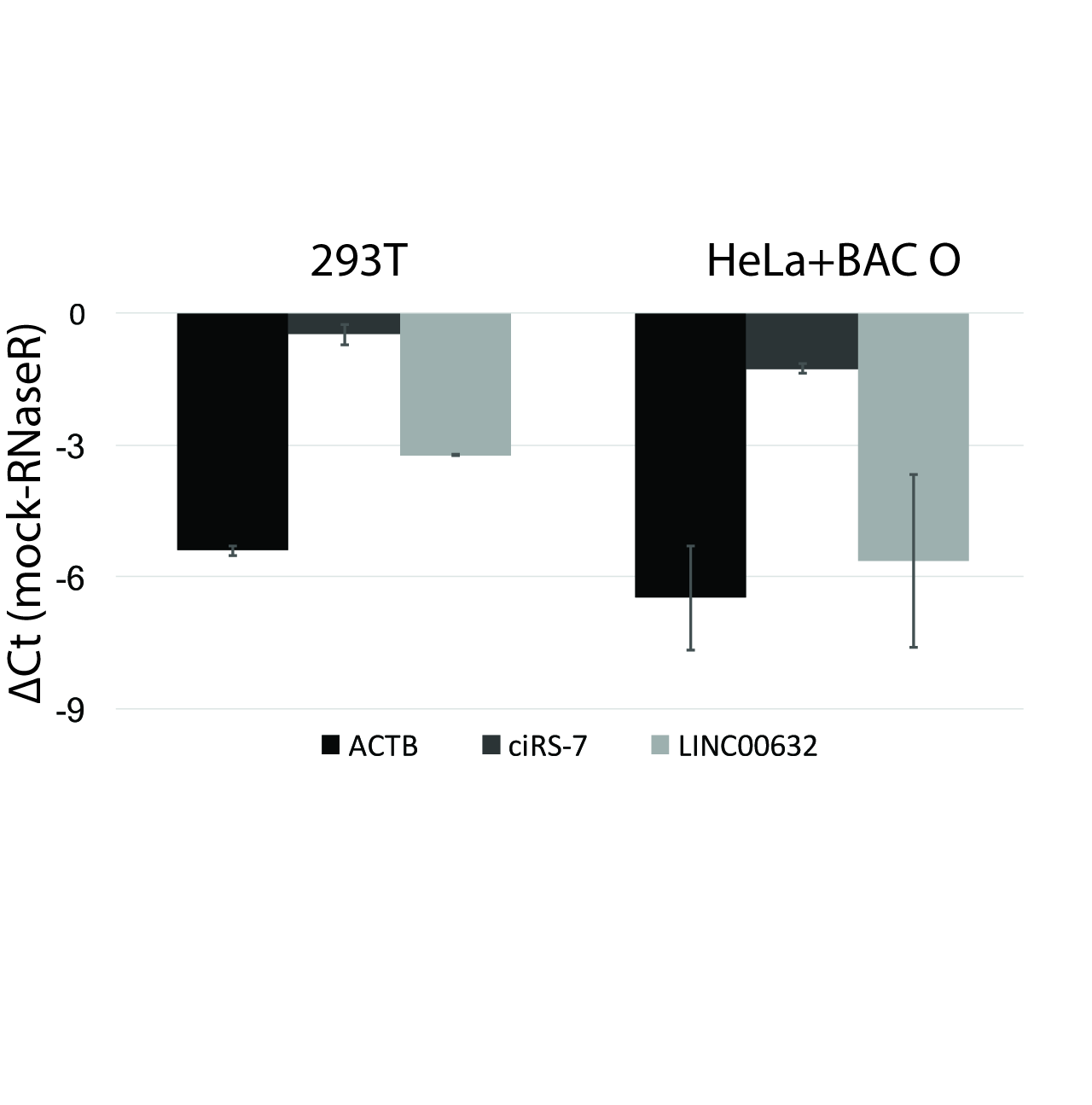

Supplement: S5 Fig — LINC00632 isoform T3 was measured in both cases. Error bars represent the standard deviation of biological replicates. (TIF) [file pgen.1007114.s005.tif]

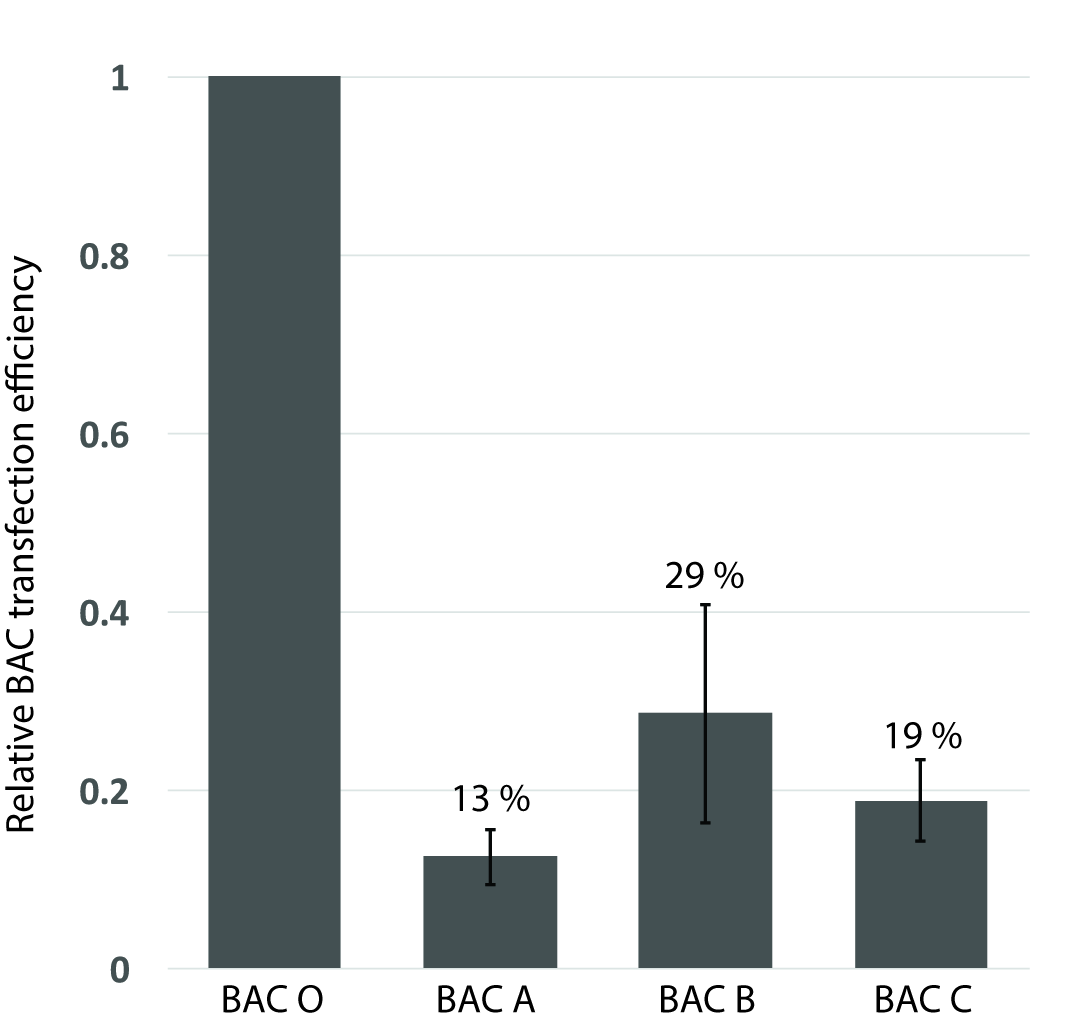

Supplement: S6 Fig — Error bars represent the standard deviation of biological replicates (with error propagated from BAC O). (TIF) [file pgen.1007114.s006.tif]

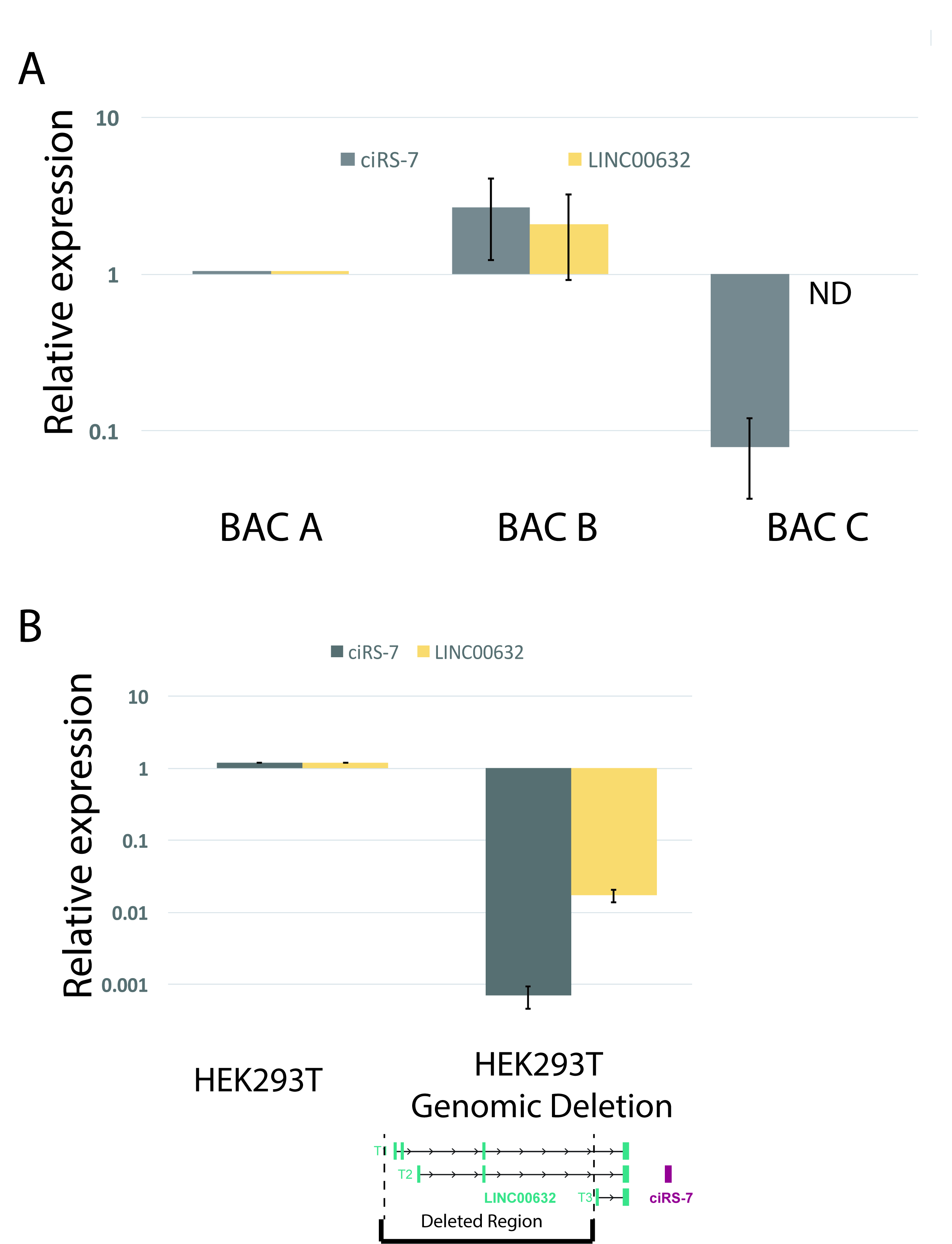

Supplement: S7 Fig — (A) RNA expression in HeLa cells transfected with BACs A, B, and C. All values have been normalized to those for BAC A, and error bars represent the standard deviation of biological replicates (with error propagated from BAC A). (B) RNA expression of isoforms in wild-type HEK293T and in a cloned strain of HEK293T in which the putative ciRS-7 promoters have been deleted. (TIF) [file pgen.1007114.s007.tif]

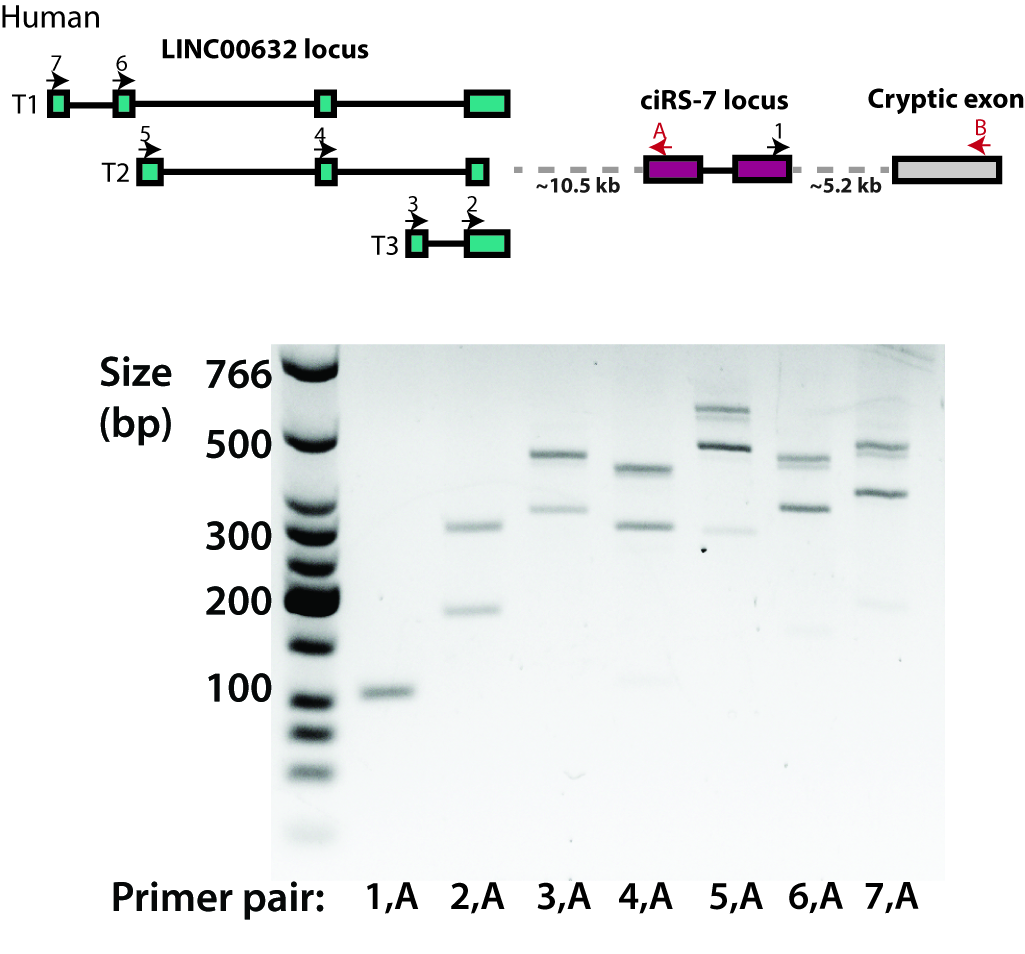

Supplement: S8 Fig — (TIF) [file pgen.1007114.s008.tif]

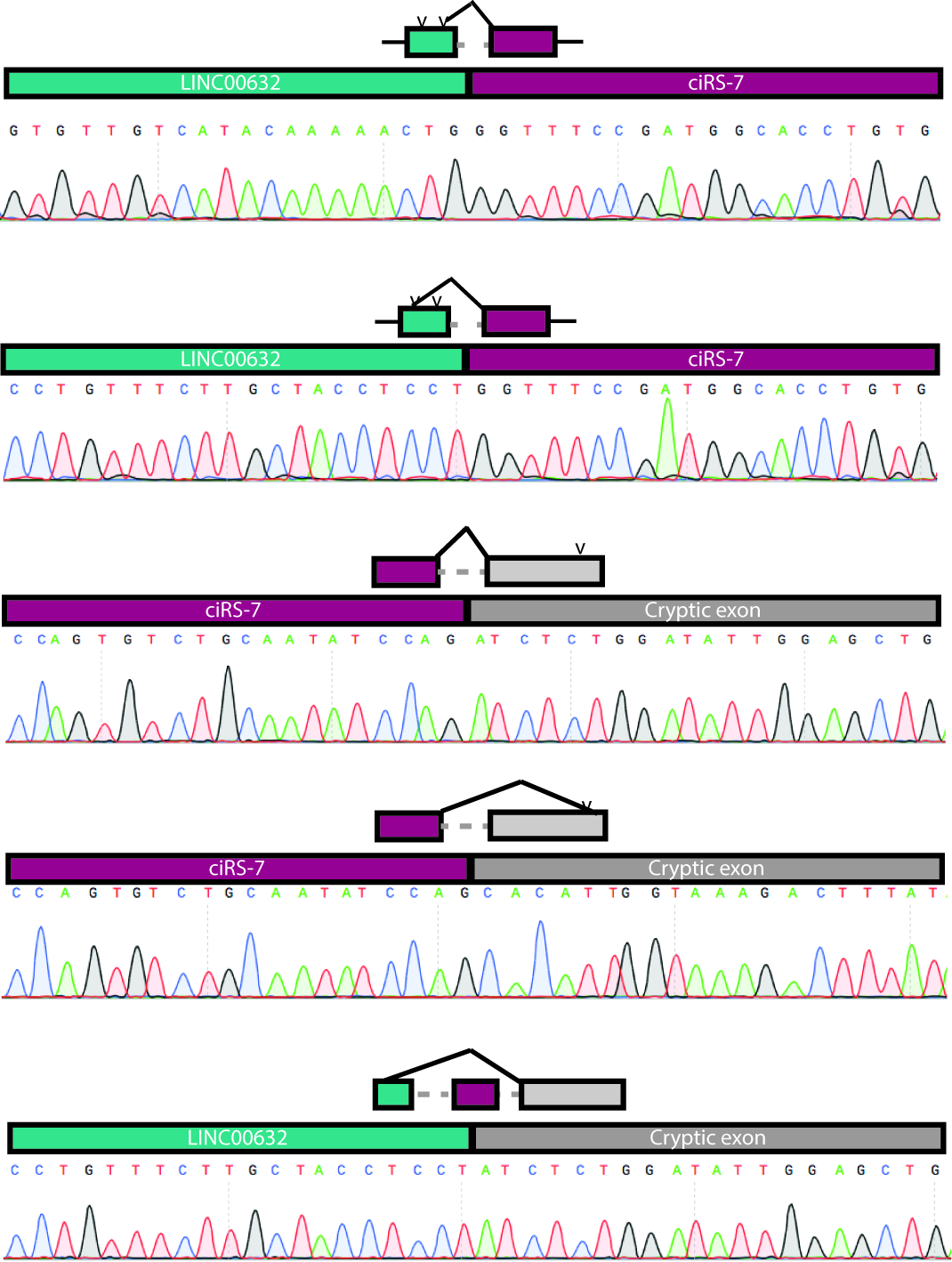

Supplement: S9 Fig — (TIF) [file pgen.1007114.s009.tif]

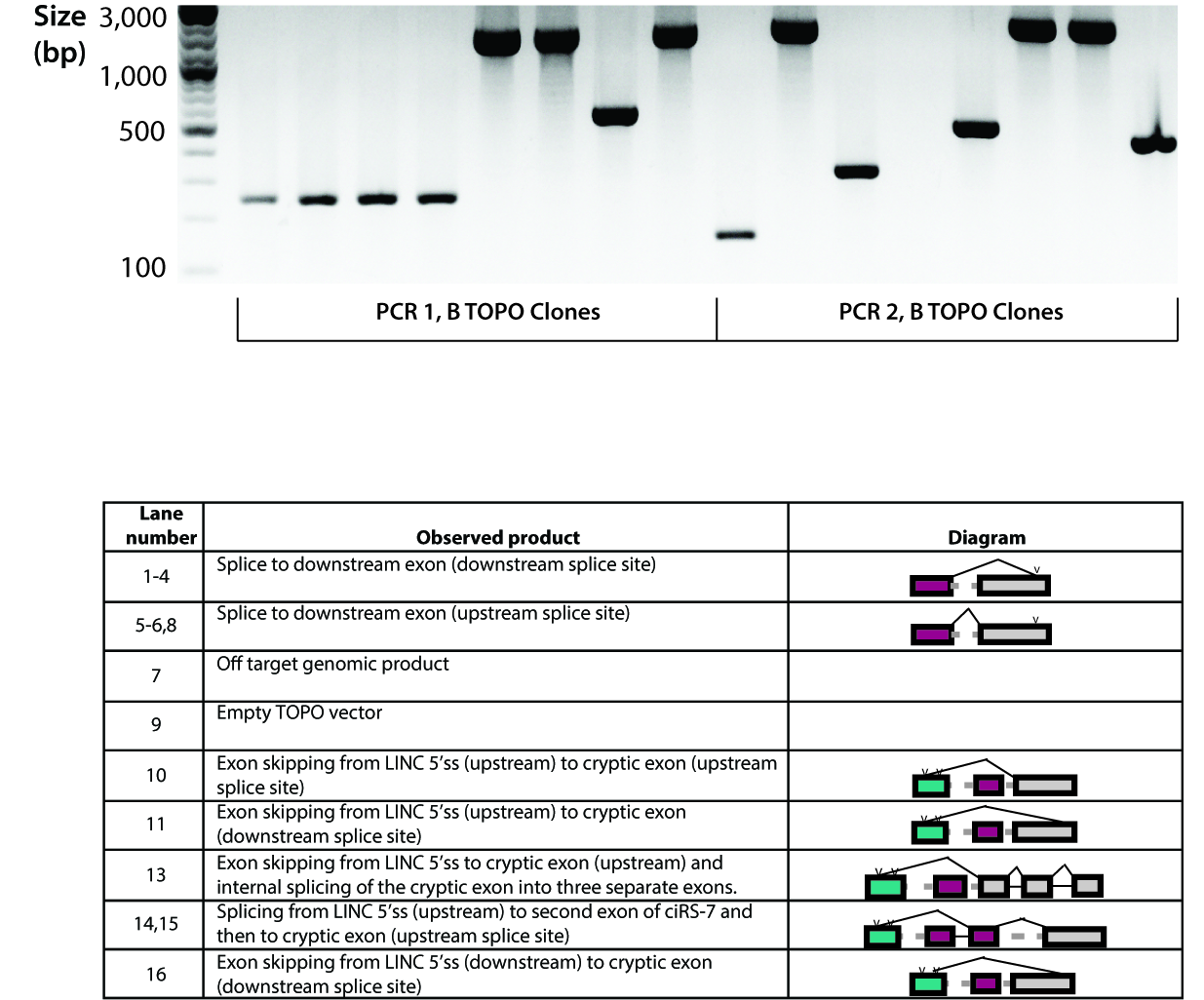

Supplement: S10 Fig — (TIF) [file pgen.1007114.s010.tif]

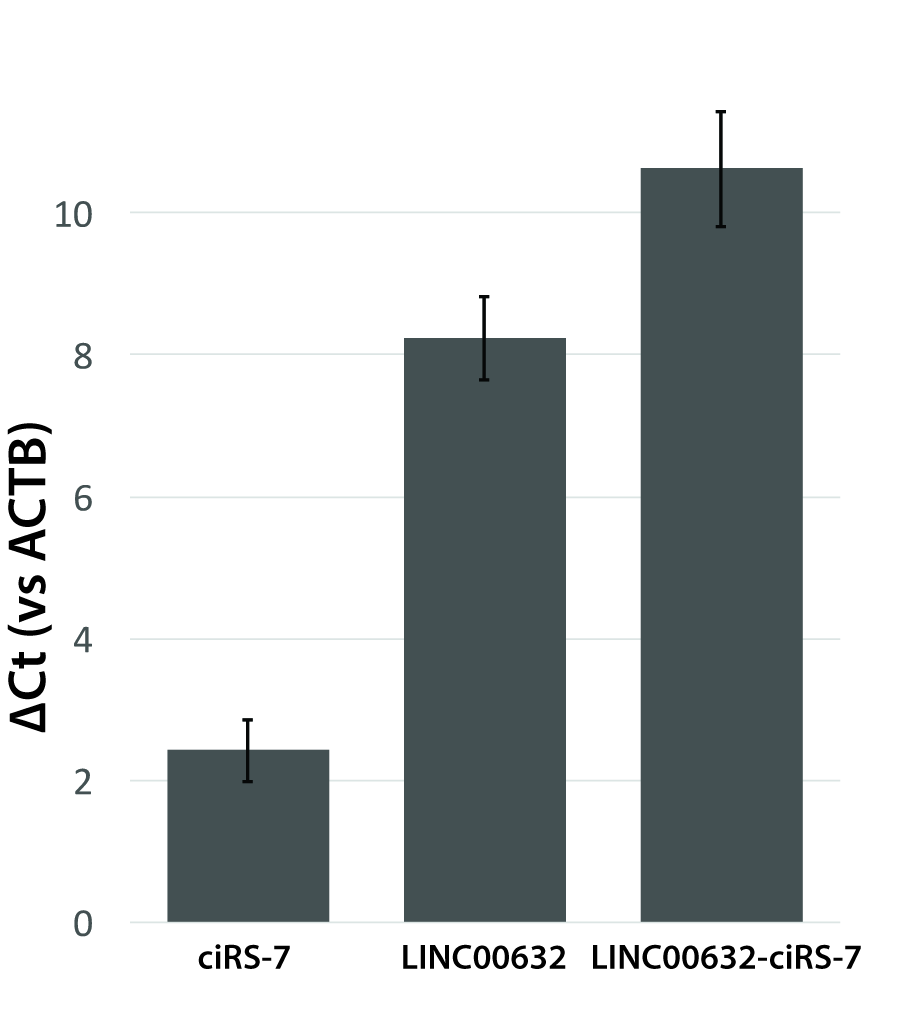

Supplement: S11 Fig — Higher values indicate lower expression. Error bars represent standard deviation of biological replicates. (TIF) [file pgen.1007114.s011.tif]

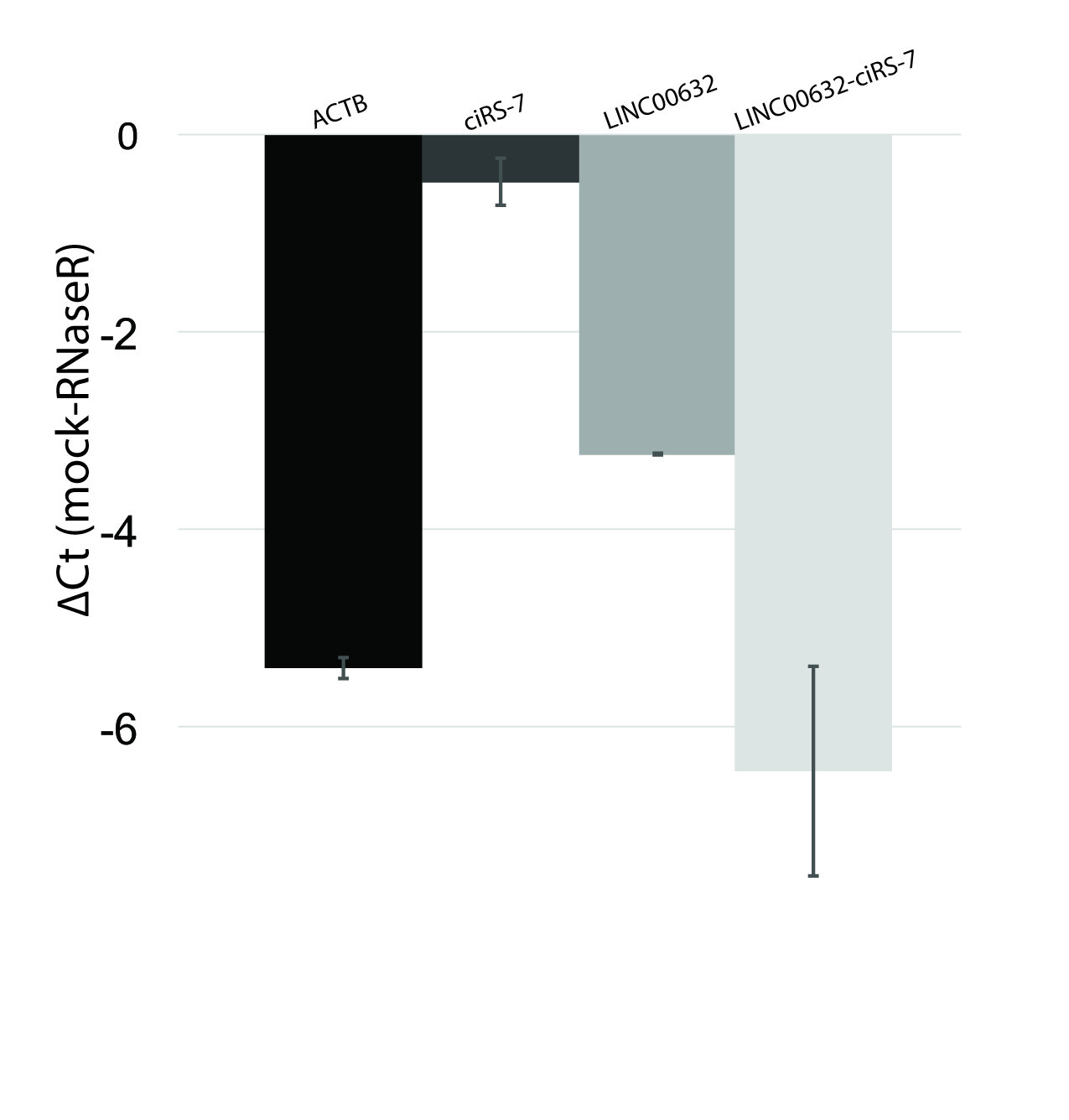

Supplement: S12 Fig — (TIF) [file pgen.1007114.s012.tif]

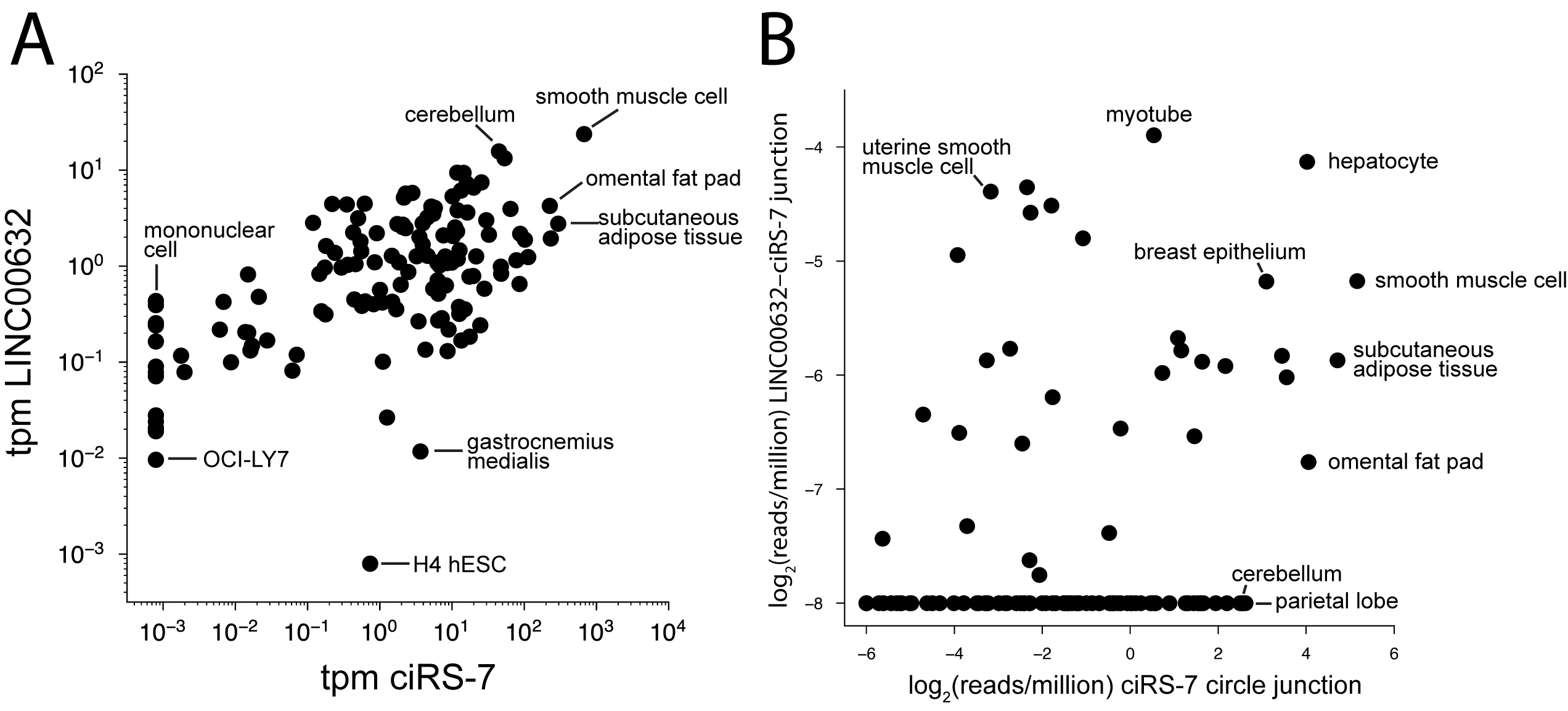

Supplement: S13 Fig — (A) LINC00632 and ciRS-7 gene-level quantification, transcripts per million reads (tpm) (B) ciRS-7 backsplice and LINC00632-ciRS-7 junctional counts per million reads. (TIF) [file pgen.1007114.s013.tif]

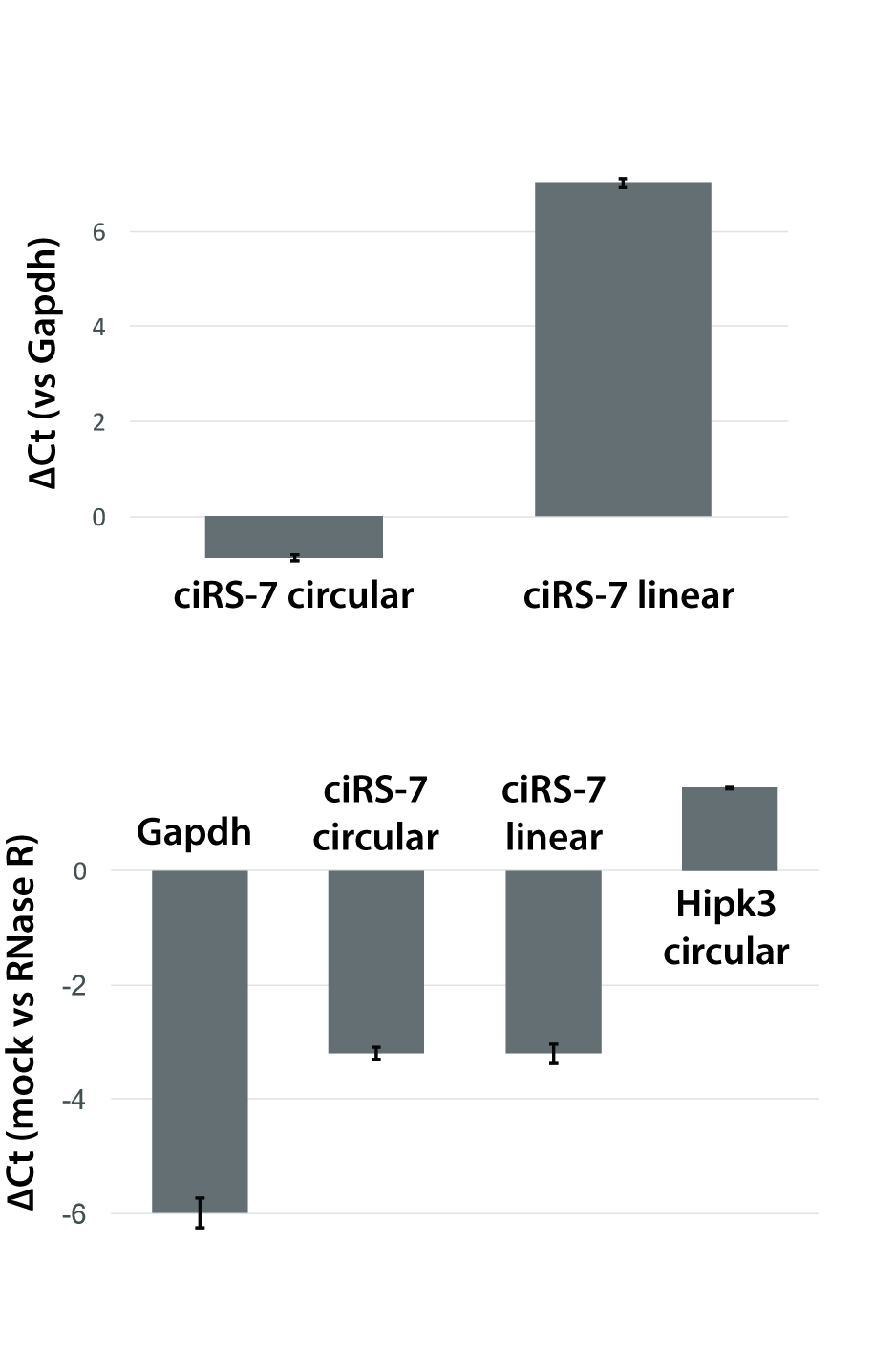

Supplement: S14 Fig — Error bars represent standard deviation of technical replicates. (TIF) [file pgen.1007114.s014.tif]

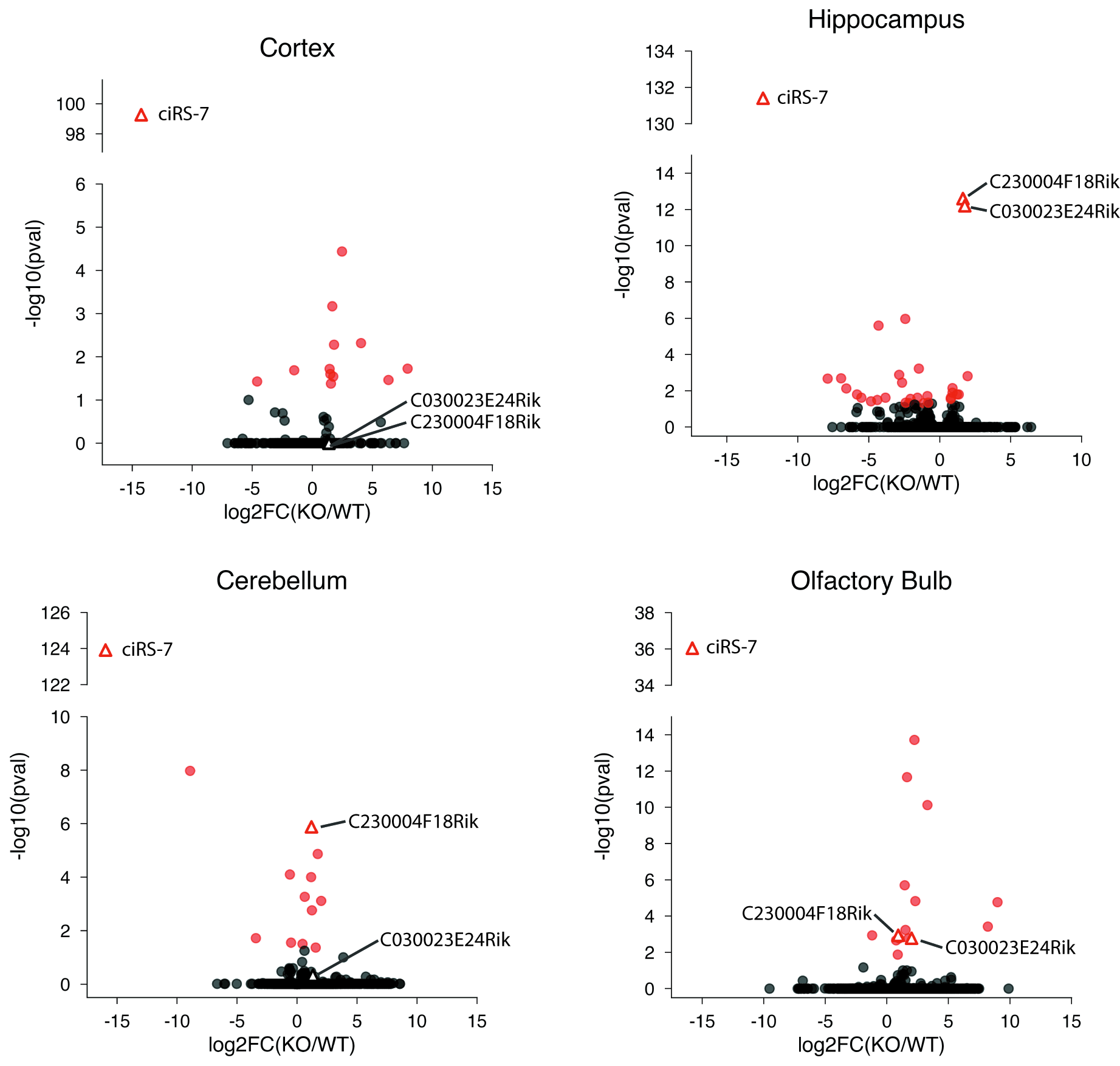

Supplement: S15 Fig — (TIF) [file pgen.1007114.s015.tif]

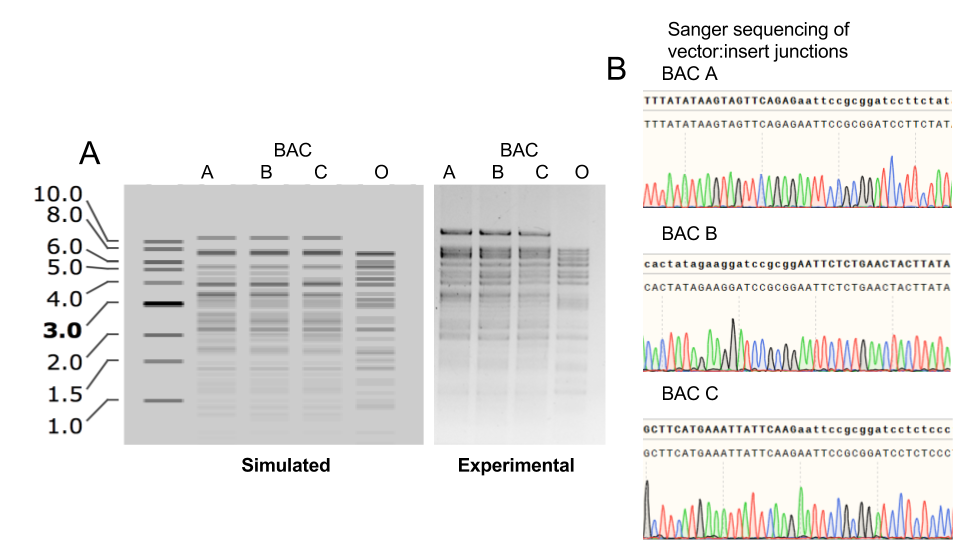

Supplement: S16 Fig — (A) Simulated and experimental BanI digest of the four BACs used in this study. The agreement of these footprints supports that BAC inserts are as reported and have not been significantly altered by bacterial recombination. (B) Sanger sequencing of the 5’ ends of BAC genomic inserts. The vector sequence is in lowercase; the genomic insert sequence is in uppercase. (TIF) [file pgen.1007114.s016.tif]
