# Supplementary material for: ciRS-7 exonic sequence is embedded in a long non-coding RNA locus
Source: PLoS Genet. 2017 Dec 13;13(12):e1007114. doi: 10.1371/journal.pgen.1007114 (PMC5745005; doi:10.1371/journal.pgen.1007114)
Supplement: S2 File — (PDF) [file pgen.1007114.s018.pdf]

```
# perl script to split reads from  unaligned KNIFE output

$myflag=" ";
$genome="mm10";
$readir="/*/*/*/*/KNIFE_dirs/knife_outputs/mouse_AgoRIP_SRR1785046/orig/unaligned/";
 $stem="mouse_rip";

$genome="hg19";
$readir="/*/*/*/*/*/knife_out/H1ESC_01192017/orig/unaligned/";
$stem="H1ESC_find";

$remake=1;
$mysize=20;
if ($remake==1){
system ("rm read1_".$stem);
system ("rm read2_".$stem);

#opendir (DIR, $readir);
$fastq=1;
$prefix="";
$fastq=0;
$prefix=">";

$offset=50; # where to start the pseudo read 2
#foreach my $fp (glob("$readir/*fasta")) {
          print "readdir is $readir\n";
   foreach my $fp (glob("$readir/*fasta")) {

## code splits reads into kmers
print "$fp\n";
open (F,$fp);
open (R1,">>read1_".$stem);
open (R2,">>read2_".$stem);

$i=0;
while ($he=<F>){
$i=$i+1;
chomp($he);
$he2=substr($he,0,$mysize);
$h=<F>;
chomp($h);
          $myl=length($h);
          # take 20mers;
          $mysb1=substr($h,0,$mysize);
```

```
                        $mysb2=substr($h,$offset,$mysize);
#print "$h\n$mysb1\t$mysb2\n";
if (length($mysb1)>0 & length($mysb2)>0 ){
                print R1 $prefix.$he."_".$i."_read_5\n$mysb1\n";
                print R2 $prefix.$he."_".$i."_read_3\n$mysb2\n";

                        $d=<F>;
                if ($fastq==1){
                        print R1 "$d\n";
                        print R2 "$d\n";
}
                        $d=<F>;
                if ($fastq==1){
                        $d2=substr($d,0,$mysize);
                        print R1 "$d2\n";
                        print R2 "$d2\n";
}

#           print  ">".$i."_read_5\n$mysb1\n";
#               print  ">".$i."_read_3\n$mysb2\n";
            }
    }

}
}

if (!($fastq)) {$myflag=" -f ";}


$cgenome=""; # for mouse
$cgenome= "human_"; # for human
system (" bowtie2  -p 8  ".$myflag." -x kalisto_index/".$cgenome."Cdr1_up_down_100k
read2_".$stem." > orphanbowtie_2_cdr1_".$stem);

system( " bowtie2  -p 8  ".$myflag." -x kalisto_index/".$cgenome."Cdr1_up_down_100k
read1_".$stem." > orphanbowtie_1_cdr1_".$stem);
```

```
##R SCRIPT

library(data.table)

## processing at the command line

#more orphanbowtie_2_cdr1_mouse_RIP |grep uc > uc_orphanbowtie_2_mouse_rip
#more orphanbowtie_1_cdr1_mouse_RIP |grep uc > uc_orphanbowtie_1_mouse_rip
#grep uc orphanbowtie_1_cdr1_H1ESC_find > uc_orphanbowtie_1_cdr1_H1ESC_find
#grep uc orphanbowtie_2_cdr1_H1ESC_find > uc_orphanbowtie_2_cdr1_H1ESC_find

#infile="mouse_rip"
#infile="cdr1_H1ESC_find"

for (infile in (c("cdr1_H1ESC_find", "mouse_rip"))) {
rm(myt)
#for (infile in (c("cdr1_H1ESC_find"))) {

m1=data.table(read.delim(paste("uc_orphanbowtie_1_", infile,sep=""), header=F,
skip=1)[,c(1:10)])
m2=data.table(read.delim(paste("uc_orphanbowtie_2_", infile,sep=""), header=F,
skip=1)[,c(1:10)])

## split

m1[,readid:= as.character(lapply(strsplit(paste(m1$V1), split="r"), "[", 1))]
m2[,readid:= as.character(lapply(strsplit(paste(m2$V1), split="r"), "[", 1))]

m1[,readL:=nchar(paste(m1$V10))]
m2[,readL:=nchar(paste(m2$V10))]

mq=(-1) #
lower.l=18

used=merge(m1[V5>mq & readL>lower.l,list(readid,V2,V4,V10, readL)],m2[V5>mq &
readL>lower.l,list(readid,V2,V4,V10,readL)], by="readid")

used[,V4.x:=V4.x+ -100000]
used[,V4.y:=V4.y-100000]

#table for for human ESC

if (infile %like% "H1"){
```

```
myt=used[(abs(V4.y)<2000| abs(V4.x)<2000 )& V2.x==V2.y & abs(V4.x-V4.y)>1000][order(-
abs(abs(V4.x-V4.y))), list(V4.x,V4.y, V4.x-V4.y, V10.x, V10.y, readL.x,readL.y,readid)]
}

#for mouse
if (infile %like% "mouse"){

myt=used[(abs(V4.y)<2500| abs(V4.x)<2500 )& V2.x==V2.y & abs(V4.x-V4.y)>1000][order(-
abs(abs(V4.x-V4.y))), list(V4.x,V4.y, V4.x-V4.y, V10.x, V10.y, readL.x,readL.y,readid)][V4.x<V4.y]


## with T appended, new circle splicing

#>
myt2=used[abs(V4.x)<1000 & V4.y<(-500)& (V4.x)<(-500 )& V2.x==V2.y & abs(V4.x-
V4.y)>500][order(-abs(abs(V4.x-V4.y))), list(V4.x,V4.y, V4.x-V4.y, V10.x, V10.y,
readL.x,readL.y,readid)]


#top reads are linear isoforms
#>

myt=cbind(myt,myt2)


}
 names(myt)[1:5]=c("offset_1","offset_2","absdiff","seq1","seq2")

write.table(file=paste(infile,"_Routput.tab", sep=""),myt[,1:8], sep="\t", quote=F ,
row.names=F)
}
```

HUMAN OUTPUT

(Offsets are with respect to the annotated ciRS-7 transcript)

READ-id	offset_1	offset_2	absdiff	seq1	seq2	Splice
>SRR5048129.92905492_2774860_	25	-10841	10866	GTCTTCCAACAACTCCGGGC	CACACGTGTTGTCATACAAA	**Novel Linear RNA**
>SRR5048080.59411858_2626289_	1443	7835	-6392	GATCTTCTGACATTCAGGTC	TAAAGACTTTATAACTTCCA	**Novel Linear RNA**
>SRR5048080.100875739_3206966_	21	1456	-1435	CAAGGTCTTCCAACAACTCC	TCAGGTCTTCCAGTGTCTGC	**ciRS-7 circle**
>SRR5048080.103424773_5101672_	1457	22	1435	CAGGTCTTCCAGTGTCTGCA	AAGGTCTTCCAACAACTCCG	**ciRS-7 circle**
>SRR5048080.103561152_236718_	12	1447	-1435	CACCTGTGTCAAGGTCTTCC	TTCTGACATTCAGGTCTTCC	**ciRS-7 circle**
>SRR5048080.104022166_1418013_	1443	8	1435	GATCTTCTGACATTCAGGTC	ATGGCACCTGTGTCAAGGTC	**ciRS-7 circle**
>SRR5048080.17777487_4616291_	23	1458	-1435	AGGTCTTCCAACAACTCCGG	AGGTCTGCCAGTGTCTGCAA	**ciRS-7 circle**
>SRR5048080.22632841_6033667_	24	1459	-1435	GGTCTTCCAACAACTCAGGG	GGTCTTCCAGTGTCTGCAAT	**ciRS-7 circle**
>SRR5048080.24019860_2118332_	22	1457	-1435	AAGGTCTTCCAACAACTCCG	CAGGTCTTCCAGTGTCTGCA	**ciRS-7 circle**
>SRR5048080.25232605_5539467_	1449	14	1435	CTGACATTCAGGTCTTCCAG	CCTGTGTCAAGGTCTTCCAA	**ciRS-7 circle**
>SRR5048080.25619456_2851736_	12	1447	-1435	CACCTGTGTCAAGGTCTTCC	TTCTGACATTCAGGTCTTCC	**ciRS-7 circle**
>SRR5048080.26343594_3864290_	16	1451	-1435	TGTGTCAAGGTCTTCCAACA	GACATTCAGGTCTTCCAGTG	**ciRS-7 circle**
>SRR5048080.33788535_4485555_	25	1460	-1435	GTCTTCCAACAACTCCGGGC	GTCTTCCAGTGTCTGCAATA	**ciRS-7 circle**
>SRR5048080.34113819_4397163_	27	1462	-1435	CTTCCAACAACTCCGGGTCT	CTTCCAGTGTCTACAATATC	**ciRS-7 circle**
>SRR5048080.40020025_3937366_	1447	12	1435	TTCTGACATTCAGGTCTTCC	CACCTGTGTCAAGGTCTTCC	**ciRS-7 circle**
>SRR5048080.40408537_5095838_	1433	-2	1435	CCAGTGTGCTGATCTTCTGA	AGGGTTTCCGATGGCACCTG	**ciRS-7 circle**
>SRR5048080.41137295_36171_	-10	1425	-1435	CAATATCCAGGGTTTCCGAT	CAACGTCTCCAGTGTGCTGA	**ciRS-7 circle**
>SRR5048080.52818665_2370515_	1442	7	1435	TGATCTTCTGACATTCAGGT	GATGGCACCTGTGTCAAGGT	**ciRS-7 circle**
>SRR5048080.57104984_1008060_	24	1459	-1435	GGTCTTCCAACAACTCCGGG	GGTCTTCCAGTGTCTGCAAT	**ciRS-7 circle**
>SRR5048080.57671465_827516_	1443	8	1435	GATCTTCTGACATTCAGGTC	ATGGCACCTGTGTCAAGGTC	**ciRS-7 circle**
>SRR5048080.59527248_6020342_	1464	29	1435	TCCAGTGTCTGCAATATCCA	TCCAACAACTCCGGGTCTTC	**ciRS-7 circle**
>SRR5048080.60338221_336699_	1436	1	1435	GTGTGCTGATCTTCTGACAT	GTTTCCGATGGCACCTGTGT	**ciRS-7 circle**
>SRR5048080.60742841_4434991_	13	1448	-1435	ACCTGTGTCAAGGTCTTCCA	TCTGACATTCAGGTCTTCCA	**ciRS-7 circle**
>SRR5048080.60767964_3939516_	1447	12	1435	TTCTGACATTCAGGTCTTCC	CACCTGTTTCAAGGTCTTCC	**ciRS-7 circle**
>SRR5048080.65042132_5189714_	25	1460	-1435	GTCTTCCAACAACTCCGGGC	GTCTTCCAGTGTCTGCAATA	**ciRS-7 circle**
>SRR5048080.6766034_3745788_	16	1451	-1435	TGTGTCAAGGTCTTCCAACA	GACATTCAGGTCTTCCAGTG	**ciRS-7 circle**
>SRR5048080.68598631_2481961_	32	1467	-1435	AACAACTCCGGGTCTTCCAG	AGTGTCTGCAATATCCAGGG	**ciRS-7 circle**
>SRR5048080.70252381_1985001_	25	1460	-1435	GTCTTCCAACAACTCCGGGC	GTCTTCCAGTGTCTGCAATA	**ciRS-7 circle**
>SRR5048080.76978837_5758561_	15	1450	-1435	CTGTGTCAAGGTCTTCCAAC	TGACATTCAGGTCTTCCAGT	**ciRS-7 circle**
>SRR5048080.77628051_5856711_	1440	5	1435	GCTGATCTTCTGACATTCAG	CCGATGGCACCTGTGTCAAG	**ciRS-7 circle**
>SRR5048080.80443644_1003727_	1439	4	1435	TGCTGATCTTCTGACATTCA	TCCNATGGCACCTGTGTCAA	**ciRS-7 circle**
>SRR5048080.81980405_4719362_	1448	13	1435	TCTGACATTCAGGTCTTCCA	ACCTGTGTCAAGGTCTTCCA	**ciRS-7 circle**
>SRR5048080.83748541_4352065_	25	1460	-1435	GTCTTCCAACAACTCCGGGC	GTCTTCCAGTGTCTGCAATA	**ciRS-7 circle**
>SRR5048080.87512496_5740796_	1446	11	1435	CTTCTGACATTCAGGTCTTC	GCACCTGTGTCAAGGTCTTC	**ciRS-7 circle**
>SRR5048080.88581756_1524552_	1439	4	1435	TGCTGATCTTCTGACATTCA	TCCGATGGCACCTGTGTCAA	**ciRS-7 circle**
>SRR5048080.92538225_262888_	21	1456	-1435	CAAGGTCTTCCAACAACTCC	TCAGGTCTTCCAGTGTCTGC	**ciRS-7 circle**
>SRR5048080.94482953_3613367_	27	1462	-1435	CTTCCAACAACTCCGGGTCT	CTTCCAGTGTCTGCAATATC	**ciRS-7 circle**
>SRR5048080.94785784_4487883_	16	1451	-1435	TGTGTCAAGGTCTTCCAACA	GACATTCAGGTCTTCCAGTG	**ciRS-7 circle**
>SRR5048129.12555659_2744061_	1431	-4	1435	CTCCAGTGTGCTGATCTTCT	CCAGGGTTTCCGATGGCACC	**ciRS-7 circle**
>SRR5048129.13833719_1834963_	-10	1425	-1435	CAATATCCAGGGTTTCCGAT	CAACGTCTCCAGTGTGCTGA	**ciRS-7 circle**
>SRR5048129.17755068_3311121_	-10	1425	-1435	CAATATCCAGGGTTTCCGAT	CAACGTCTCCAGTGTGCTGA	**ciRS-7 circle**
>SRR5048129.18893587_1058036_	22	1457	-1435	AAGGTCTTCCAACAACTCCG	CAGGTCTTCCAGTGTCTGCA	**ciRS-7 circle**
>SRR5048129.27996523_1639205_	1431	-4	1435	CTCCAGTGTGCTGATCTTCT	CCAGGGTTTCCGATGGCACC	**ciRS-7 circle**
>SRR5048129.28487901_2895202_	1431	-4	1435	CTCCAGTGTGCTGATCTTCT	CCAGGGTTTCCGATGGCACC	**ciRS-7 circle**
>SRR5048129.29856715_1306793_	1438	3	1435	GTGCTGATCTTCTGACATTC	TTCCGATGGCACCTGTGTCA	**ciRS-7 circle**
>SRR5048129.30001069_385713_	23	1458	-1435	AGGTCTTCCAACAACTCCGG	AGGTCTTCCAGTGTCTGCAA	**ciRS-7 circle**
>SRR5048129.31791353_1097441_	2	1437	-1435	TTTCCGATGGCACCTGTGTC	TGTGCTGATCTTCTGACATT	**ciRS-7 circle**
>SRR5048129.35480370_2522009_	-2	1433	-1435	AGGGTTTCCGATGGCACCAG	CCAGTGTGCTGATCTTCTGA	**ciRS-7 circle**
>SRR5048129.3892134_564946_	-8	1427	-1435	ATATCCAGGGTTTCCGATGG	ACGTCTCCAGTGTGCTGATC	**ciRS-7 circle**
>SRR5048129.41692879_1636627_	23	1458	-1435	AGGTCTTCCAACAACTCCGG	AGGTCTTCCAGTGTCTGCAA	**ciRS-7 circle**
>SRR5048129.42238903_1974507_	-3	1432	-1435	CAGGGTTTCCGATGGCACCT	TCCAGTGTGCTGATCTTCTG	**ciRS-7 circle**
>SRR5048129.4292261_2644040_	23	1458	-1435	AGGTCTTCCAACAACTCCGG	AGGTCTTCCAGTGTCTGCAA	**ciRS-7 circle**
>SRR5048129.4486553_128998_	14	1449	-1435	CCTGTGTCAAGGTCTTCCAA	CTGACATTCAGGTCTTCCAG	**ciRS-7 circle**
>SRR5048129.47675279_3081710_	30	1465	-1435	CCAACAACTCCGGGTCTTCC	CCAGTGTCTGCAATATCCAG	**ciRS-7 circle**
>SRR5048129.82072305_1315148_	25	1460	-1435	GTCTTCCAACAACTCCGGGT	GTCTTCCAGTGTCTGCAATA	**ciRS-7 circle**
>SRR5048129.84877420_3001719_	20	1455	-1435	TCAAGGTCTTCCAACAACTC	TTCAGGTCTTCCAGTGTCTG	**ciRS-7 circle**

>SRR5048129.88921537_583804_	-9	1426	-1435	AATATCCAGGGTTTCCGATG	AACGTCTCCAGTGTGCTGAT	**ciRS-7 circle**
>SRR5048080.4741543_1944104_	343	1442	-1099	AAAATCTGTGTCTTCCACCA	TGATCTTCTGACATTCAGGT	

MOUSE OUTPUT

(Offsets are with respect to the annotated CDR1 sense transcript)

READ-id	offset_1	offset_2	absdiff	seq1	seq2	Splice
@SRR1785046.15164429	658	85879	-85221	TGGGAAGACTTGGACTTCTG	AAAAAAAAAAAAAAAAAAAA	
@SRR1785046.15164429	-73655	658	-74313	AAAAAAAAAAAAAAAAAAAA	TGGGAAGACTTGGACTTCTG	
@SRR1785046.8863940	-18221	2288	-20509	ATTTACAGTTTCAGAGGAGG	TTGGTACTGGCACCACTGG	**Novel circRNA**
@SRR1785046.13497132	-18219	2290	-20509	TTACAGTTTCAGAGGAGGAG	GGTACTGGCACCACTGGAA	**Novel circRNA**
@SRR1785046.11591060	-1113	5582	-6695	TTGTTATTATTATTATTATT	AATAATAATAATAATAACAA	
@SRR1785046.18577289	-1122	5556	-6678	TTATTATTATTGTTATTATT	ATAATAACAATAATAATAA	
@SRR1785046.14815206	-617	2281	-2898	CCCTGGATACGGCAGACACC	GAAGACCTTGGTACTGGCA	**ciRS-7 circle**
@SRR1785046.4033180	-617	2281	-2898	CCCTGGATACGGCAGACACC	GAAGACCTTGGTACTGGCA	**ciRS-7 circle**
@SRR1785046.295357	-616	2282	-2898	CCTGGATACGGCAGACACCA	AAGACCTTGGTACTGGCAC	**ciRS-7 circle**
@SRR1785046.11903117	-614	2284	-2898	TGGATACGGCAGACACCAGA	GACCTTGGTACTGGCACCA	**ciRS-7 circle**
@SRR1785046.1735563	-614	2284	-2898	TGGATACGGCAGACACCAGA	GACCTTGGTACTGGCACCA	**ciRS-7 circle**
@SRR1785046.19719917	-614	2284	-2898	TGGATACGGCAGACACCAGA	GACCTTGGTACTGGCACCA	**ciRS-7 circle**
@SRR1785046.20420244	-614	2284	-2898	TGGATACGGCAGACACCAGA	GACCTTGGTACTGGCACCA	**ciRS-7 circle**
@SRR1785046.5706212	-614	2284	-2898	TGGATACGGCAGACACCAGA	GACCTTGGTACTGGCACCA	**ciRS-7 circle**
@SRR1785046.7571323	-614	2284	-2898	TGGATACGGCAGACACCAGA	GACCTTGGTACTGGCACCA	**ciRS-7 circle**
@SRR1785046.15092498	-613	2285	-2898	GGATACGGCAGACACCAGAA	ACCTTGGTACTGGCACCAC	**ciRS-7 circle**
@SRR1785046.15495250	-613	2285	-2898	GGATACGGCAGACACCAGAC	ACCTTGGTACTGGCACCAC	**ciRS-7 circle**
@SRR1785046.17805708	-613	2285	-2898	GGATACGGCAGACACCAGAA	GCCTTGGTACTGGCACCAC	**ciRS-7 circle**
@SRR1785046.15887301	-608	2290	-2898	CGGCAGACACCAGAAAACCT	GGTACTGGCACCACTGGAA	**ciRS-7 circle**
@SRR1785046.15598539	-607	2291	-2898	GGCAGACACCAGAAAACCTG	GTACTGGCACCACTGGAAA	**ciRS-7 circle**
@SRR1785046.16084222	-607	2291	-2898	GGCAGACACCAGAAAACCTG	GTACTGGCACCACTGGAAA	**ciRS-7 circle**
@SRR1785046.3012701	-606	2292	-2898	GCAGACACCAGAAAACCTGA	TACTGGCACCACTGGAAAC	**ciRS-7 circle**
@SRR1785046.3764585	-606	2292	-2898	GCAGACACCAGAAAACCTGA	TACTGGCACCACTGGAAAC	**ciRS-7 circle**
@SRR1785046.11337835	-605	2293	-2898	CAGACACCAGAAAACCTGAA	ACTGGCACCACTGGAAACC	**ciRS-7 circle**
@SRR1785046.3469567	-605	2293	-2898	CAGACACCAGAAAACCTGAA	ACTGGCACCACTGGAAACC	**ciRS-7 circle**
@SRR1785046.10735139	-602	2296	-2898	ACACCAGAAAACCTGAATGT	GGCACCACTGGAAACCCTG	**ciRS-7 circle**
@SRR1785046.13906927	-602	2296	-2898	ACACCAGAAAACCTGAATGT	GGCACCACTGGAAACCCTG	**ciRS-7 circle**
@SRR1785046.9999855	-602	2296	-2898	ACACCAGAAAACCTGAATGT	GGCACCACTGGAAACCCTG	**ciRS-7 circle**
@SRR1785046.2358671	-601	2297	-2898	CACCAGAAAACCTGAATGTC	GCACCACTGGAAACCCTGG	**ciRS-7 circle**
@SRR1785046.9906804	-601	2297	-2898	CACCAGAAAACCTGAATGTC	GCACCACTGGAAACCCTGG	**ciRS-7 circle**
@SRR1785046.10904736	-600	2298	-2898	ACCAGAAAACCTGAATGTCA	CACCACTGGAAACCCTGGA	**ciRS-7 circle**
@SRR1785046.20378296	-600	2298	-2898	ACCAGAAAACCTGAATGTCA	CACCACTGGAAACCCTGGA	**ciRS-7 circle**
@SRR1785046.5451982	-600	2298	-2898	ACCAGAAAACCTGAATGTCA	CACCACTGGAAACCCTGGA	**ciRS-7 circle**
@SRR1785046.8440302	-600	2298	-2898	ACCAGAAAACCTGAATGTCA	CACCACTGGAAACCCTGGA	**ciRS-7 circle**
@SRR1785046.10661832	-599	2299	-2898	CCAGAAAACCTGAATGTCAA	ACCACTGGAAACCCTGGAT	**ciRS-7 circle**
@SRR1785046.11556819	-599	2299	-2898	CCAGAAAACCTGAATGTCAA	ACCACTGGAAACCCTGGAT	**ciRS-7 circle**
@SRR1785046.14371276	-599	2299	-2898	CCAGAAAACCTGAATGTCAA	ACCACTGGAAACCCTGGAT	**ciRS-7 circle**
@SRR1785046.15933337	-599	2299	-2898	CCAGAAAACCTGAATGTCAA	ACCACTGGAAACCCTGGAT	**ciRS-7 circle**
@SRR1785046.17830265	-599	2299	-2898	CCAGAAAACCTGAATGTCAA	ACCACTGGAAACCCTGGAT	**ciRS-7 circle**
@SRR1785046.2616745	-599	2299	-2898	CCAGAAAACCTGAATGTCAA	ACCACTGGAAACCCTGGAT	**ciRS-7 circle**
@SRR1785046.20519446	-598	2300	-2898	CAGAAAACCTGAATGTCAAA	CCACTGGAAACCCTGGATA	**ciRS-7 circle**
@SRR1785046.21494467	-598	2300	-2898	CAGAAAACCTGAATGTCAAA	CCACTGGAAACCCTGGATA	**ciRS-7 circle**
@SRR1785046.4258213	-598	2300	-2898	CAGAAAACCTGAATGTCAAA	CCACTGGAAACCCTGGATA	**ciRS-7 circle**
@SRR1785046.1361010	-617	2280	-2897	CCCTGGATACGGCAGACACC	GGAAGACCTTGGTACTGGCA	**ciRS-7 circle**
@SRR1785046.14961031	-617	2280	-2897	CCCTGGATACGGCAGACACC	GGAAGACCTTGGTACTGGCA	**ciRS-7 circle**
@SRR1785046.15399484	-617	2280	-2897	CCCTGGATACGGCAGACACC	GGAAGACCTTGGTACTGGCA	**ciRS-7 circle**
@SRR1785046.18722677	-617	2280	-2897	CCCTGGATACGGCAGACACC	GGAAGACCTTGGTACTGGCA	**ciRS-7 circle**
@SRR1785046.21578930	-617	2280	-2897	CCCTGGATACGGCAGACACC	GGAAGACCTTGGTACTGGCA	**ciRS-7 circle**
@SRR1785046.9423738	-617	2280	-2897	CCCTGGATACGGCAGACACC	GGAAGACCTTGGTACTGGCA	**ciRS-7 circle**
@SRR1785046.4152384	-616	2281	-2897	CCTGGATACGGCAGACACCA	GAAGACCTTGGTACTGGCAC	**ciRS-7 circle**
@SRR1785046.19867856	-615	2282	-2897	CTGGATACGGCAGACACCAG	AAGACCTTGGTACTGGCACC	**ciRS-7 circle**

@SRR1785046.7399010	-614	2283	-2897	TGGATACGGCAGACACCAGA	AGACCTTGGTACTGGCACCA	**ciRS-7 circle**
@SRR1785046.9713030	-614	2283	-2897	TGGATACGGCAGACACCAGA	AGACCTTGGTACTGGCACCA	**ciRS-7 circle**
@SRR1785046.21011419	-613	2284	-2897	GGATACGGCAGACACCAGAA	GACCTTGGTACTGGCACCAC	**ciRS-7 circle**
@SRR1785046.14064701	-612	2285	-2897	GATACGGCAGACACCAGAAA	ACCTTGGTACTGGCACCACT	**ciRS-7 circle**
@SRR1785046.14413614	-610	2287	-2897	TACGGCAGACACCAGAAAAC	CTTGGTACTGGCACCACTGG	**ciRS-7 circle**
@SRR1785046.15149512	-610	2287	-2897	TACGGCAGACACCAGAAAAC	CTTGGTACTGGCACCACTGG	**ciRS-7 circle**
@SRR1785046.16353756	-610	2287	-2897	TACGGCAGACACCAGAAAAC	CTTGGTACTGGCACCACTGG	**ciRS-7 circle**
@SRR1785046.18912107	-610	2287	-2897	TACGGCAGACACCAGAAAAC	CTTGGTACTGGCACCACTGG	**ciRS-7 circle**
@SRR1785046.21623663	-610	2287	-2897	TACGGCAGACACCAGAAAAC	CTTGGTACTGGCACCACTGG	**ciRS-7 circle**
@SRR1785046.14620904	-608	2289	-2897	CGGCAGACACCAGAAAACCT	TGGTACTGGCACCACTGGAA	**ciRS-7 circle**
@SRR1785046.16324720	-608	2289	-2897	CGGCAGACACCAGAAAACCT	TGGTACTGGCACCACTGGAA	**ciRS-7 circle**
@SRR1785046.13871680	-607	2290	-2897	GGCAGACACCAGAAAACCTG	GGTACTGGCACCACTGGAAA	**ciRS-7 circle**
@SRR1785046.18323236	-607	2290	-2897	GGCAGACACCAGAAAACCTG	GGTACTGGCACCACTGGAAA	**ciRS-7 circle**
@SRR1785046.3719266	-607	2290	-2897	GGCAGACACCAGAAAACCTG	GGTACTGGCACCACTGGAAA	**ciRS-7 circle**
@SRR1785046.4477878	-607	2290	-2897	GGCAGACACCAGAAAACCTG	GGTACTGGCACCACTGGAAA	**ciRS-7 circle**
@SRR1785046.12301350	-606	2291	-2897	GCAGACACCAGAAAACCTGA	GTACTGGCACCACTGGAAAC	**ciRS-7 circle**
@SRR1785046.4007206	-606	2291	-2897	GCAGACACCAGAAAACCTGA	GTACTGGCACCACTGGAAAC	**ciRS-7 circle**
@SRR1785046.14339710	-604	2293	-2897	AGACACCAGAAAACCTGAAT	ACTGGCACCACTGGAAACCC	**ciRS-7 circle**
@SRR1785046.14354049	-604	2293	-2897	AGACACCAGAAAACCTGAAT	ACTGGCACCACTGGAAACCC	**ciRS-7 circle**
@SRR1785046.15743401	-604	2293	-2897	AGACACCAGAAAACCTGAAT	ACTGGCACCACTGGAAACCC	**ciRS-7 circle**
@SRR1785046.18024870	-604	2293	-2897	AGACACCAGAAAACCTGAAT	ACTGGCACCACTGGAAACCC	**ciRS-7 circle**
@SRR1785046.18342953	-604	2293	-2897	AGACACCAGAAAACCTGAAT	ACTGGCACCACTGGAAACCC	**ciRS-7 circle**
@SRR1785046.18663450	-604	2293	-2897	AGACACCAGAAAACCTGAAT	ACTGGCACCACTGGAAACCC	**ciRS-7 circle**
@SRR1785046.20914385	-604	2293	-2897	AGACACCAGAAAACCTGAAT	ACTGGCACCACTGGAAACCC	**ciRS-7 circle**
@SRR1785046.3084241	-604	2293	-2897	AGACACCAGAAAACCTGAAT	ACTGGCACCACTGGAAACCC	**ciRS-7 circle**
@SRR1785046.9329119	-604	2293	-2897	AGACACCAGAAAACCTGAAT	ACTGGCACCACTGGAAACCC	**ciRS-7 circle**
@SRR1785046.9754545	-604	2293	-2897	AGACACCAGAAAACCTGAAT	ACTGGCACCACTGGAAACCC	**ciRS-7 circle**
@SRR1785046.15322178	-603	2294	-2897	GACACCAGAAAACCTGAATG	CTGGCACCACTGGAAACCCT	**ciRS-7 circle**
@SRR1785046.5026667	-603	2294	-2897	GACACCAGAAAACCTGAATG	CTGGCACCACTGGAAACCCT	**ciRS-7 circle**
@SRR1785046.529872	-603	2294	-2897	GACACCAGAAAACCTGAATG	CTGGCACCACTGGAAACCCT	**ciRS-7 circle**
@SRR1785046.11406494	-602	2295	-2897	ACACCAGAAAACCTGAATGT	TGGCACCACTGGAAACCCTG	**ciRS-7 circle**
@SRR1785046.14078477	-602	2295	-2897	ACACCAGAAAACCTGAATGT	TGGCACCACTGGAAACCCTG	**ciRS-7 circle**
@SRR1785046.3921146	-602	2295	-2897	ACACCAGAAAACCTGAATGT	TGGCACCACTGGAAACCCTG	**ciRS-7 circle**
@SRR1785046.512597	-602	2295	-2897	ACACCAGAAAACCTGAATGT	TGGCACCACTGGAAACCCTG	**ciRS-7 circle**
@SRR1785046.7331108	-602	2295	-2897	ACACCAGAAAACCTGAATGT	TGGCACCACTGGAAACCCTG	**ciRS-7 circle**
@SRR1785046.115692	-601	2296	-2897	CACCAGAAAACCTGAATGTC	GGCACCACTGGAAACCCTGG	**ciRS-7 circle**
@SRR1785046.14546509	-601	2296	-2897	CACCAGAAAACCTGAATGTC	GGCACCACTGGAAACCCTGG	**ciRS-7 circle**
@SRR1785046.15031600	-601	2296	-2897	CACCAGAAAACCTGAATGTC	GGCACCACTGGAAACCCTGG	**ciRS-7 circle**
@SRR1785046.16560945	-601	2296	-2897	CACCAGAAAACCTGAATGTC	GGCACCACTGGAAACCCTGG	**ciRS-7 circle**
@SRR1785046.17959900	-601	2296	-2897	CACCAGAAAAACTGAATGTC	GGCACCACTGGAAACCCTGG	**ciRS-7 circle**
@SRR1785046.18529774	-601	2296	-2897	CACCAGAAAACCTGAATGTC	GGCACCACTGGAAACCCTGG	**ciRS-7 circle**
@SRR1785046.19522094	-601	2296	-2897	CACCAGAAAACCTGAATGTC	GGCACCACTGGAAACCCTGG	**ciRS-7 circle**
@SRR1785046.19573975	-601	2296	-2897	CACCAGAAAACCTGAATGTC	GGCACCACTGGAAACCCTGG	**ciRS-7 circle**
@SRR1785046.20208004	-601	2296	-2897	CACCAGAAAACCTGAATGTC	GGCACCACTGGAAACCCTGG	**ciRS-7 circle**
@SRR1785046.2431671	-601	2296	-2897	CACCAGAAAACCTGAATGTC	GGCACCACTGGAAACCCTGG	**ciRS-7 circle**
@SRR1785046.9308638	-601	2296	-2897	CACCAGAAAACCTGAATGTC	GGCACCACTGGAAACCCTGG	**ciRS-7 circle**
@SRR1785046.14831703	-600	2297	-2897	ACCAGAAAACCTGAATGTCA	GCACCACTGGAAACCCTGGA	**ciRS-7 circle**
@SRR1785046.19399803	-600	2297	-2897	ACCAGAAAACCTGAATGTCT	GCACCACTGGAAACCCTGGA	**ciRS-7 circle**
@SRR1785046.303485	-600	2297	-2897	ACCAGAAAACCTGAATGTCT	GCACCACTGGAAACCCTGGA	**ciRS-7 circle**
@SRR1785046.8453217	-600	2297	-2897	ACCAGAAAACCTGAATGTCA	GCACCACTGGAAACCCTGGA	**ciRS-7 circle**
@SRR1785046.14652499	-599	2298	-2897	CCAGAAAACCTGAATGTCAA	CACCACTGGAAACCCTGGAT	**ciRS-7 circle**
@SRR1785046.17313966	-599	2298	-2897	CCAGAAAACCTGAATGTCAA	CACCACTGGAAACCCTGGAT	**ciRS-7 circle**
@SRR1785046.20127525	-599	2298	-2897	CCAGAAAACCTGAATGTCAA	CACCACTGGAAACCCTGGAT	**ciRS-7 circle**
@SRR1785046.21624889	-599	2298	-2897	CCAGAAAACCTGAATGTCAA	CACCACTGGAAACCCTGGAT	**ciRS-7 circle**

@SRR1785046.14805258	-598	2299	-2897	CAGAAAACCTGAATGTCAAG	ACCACTGGAAACCCTGGATA	**ciRS-7 circle**
@SRR1785046.20880558	-598	2299	-2897	CAGAAAACCTGAATGTCAAG	ACCACTGGAAACCCTGGATA	**ciRS-7 circle**
@SRR1785046.11393391	-470	2253	-2723	AGACCTGGAGGTGAAGGAAG	GCAGTTGCTGGAAGACCTGG	
@SRR1785046.12846588	-322	2263	-2585	CATGGATTTGTGGAAGGCAT	GAAGACCTGGAGATGTTGG	
@SRR1785046.2991319	-358	2174	-2532	CGTAGATTTTTAGGAAGACT	GAAGACATATATCATCTGGA	
@SRR1785046.19438809	-289	2170	-2459	GACATGGATTTTCCAGAAGA	GTTGGAAGACATATATCATC	
@SRR1785046.14979869	-148	2277	-2425	AGACATGGATTTTTGGAAGG	GTTGGAAGACCTTGGTACTG	
@SRR1785046.8692546	-290	2116	-2406	AGACATGGATTTTCCAGAAG	CACGGAAGATCTAGATACCA	
@SRR1785046.6171735	-373	1970	-2343	TAGATTTGTGGAAGGCGTAG	AGACTTGGATTTTTCTGGA	
@SRR1785046.14228459	-165	2099	-2264	GGCATAGATTTTCAGGAAGA	CCAGAAGATCTAGATATCAC	
@SRR1785046.10651391	-28	2216	-2244	TTCAGGAAGATTTGGATTTT	TGATTGTCTGGAAGACCTT	
@SRR1785046.3378798	-81	1972	-2053	CACGAAGACATGGATTTTTG	ACTTGGATTTTTCAGGAAGA	
@SRR1785046.11346947	2294	3972	-1678	CTGGCACCACTGGAAACCCT	TATTACTTGGTTCTAGATC	**Novel Linear RNA**
@SRR1785046.18464861	299	1935	-1636	GGGAAGACTTGGATTTCTGG	CTTGGATTTTTCAGGAAGAC	
@SRR1785046.5391078	-175	1361	-1536	ATTTATGGAAGGCATAGATT	GGGAAGACTCGGATTTTCAG	
@SRR1785046.19254497	187	1718	-1531	TTCTGGGAAGACTCGGATTT	TAATTTTCAGGAAGACATAG	
@SRR1785046.7935027	-288	1229	-1517	ACATTGATTTTCCAGAAGAC	ATTTTCCGGAAGACTTGGAT	
@SRR1785046.4067421	-56	1445	-1501	TCGTGGATTTTTGGGAAGAT	ATTTCTGGGAAGACATGGAT	
@SRR1785046.8573577	109	1466	-1357	ACAGATTACTGGGAAGAAGT	TTCGGGAAGACTTCGATTT	
@SRR1785046.18976692	117	1473	-1356	CTGGGAAGAAGTGGATCTTT	AGACTTCGATTTCTGGGAAG	
@SRR1785046.2304252	622	1972	-1350	TGGGAAGACTTGGACTTCTG	ACTTGGATTTTTCGGGAAGA	
@SRR1785046.17888453	170	1479	-1309	TCTGGGAAGACTTGGACTTC	CGATTTCTGGGAAGACTTG	
@SRR1785046.2022621	-275	971	-1246	AGAAGACTTGGATTTCTGGG	ACTTGGATTTCTGGGAAGAC	
@SRR1785046.20683963	-60	1172	-1232	GAAGTCGTGGATTTTTGGGA	TGGATTTTCGGGAAGACTT	
@SRR1785046.10520557	-115	1086	-1201	GGAAGACTTGGATTTTCAGG	CTTCTGGGAAAACGTAGATT	
@SRR1785046.13923387	283	1461	-1178	GAAGACTCGGACTTCTGGGA	GGATTTTCGGGAAGACTTC	
@SRR1785046.7498862	165	1293	-1128	GGACTTCTGGGAAGACTTGG	AGACTTCGATTTCCCAGAAG	
@SRR1785046.7247968	480	1604	-1124	GGAAGACTTCGATTTCTGGG	GTGGATTTAATGGAAGACT	
@SRR1785046.21187901	-10	1076	-1086	TTTGGGAAGTCATGGATTTT	AAGACTTGGATTTCTGGGAA	
@SRR1785046.313192	389	1474	-1085	TGGAAGACTCGGATTTCTGG	GACTTCGATTTCTGGGAAGA	
@SRR1785046.13434682	-20	1036	-1056	GATTTGGATTTTTGGGAAGT	CAGGAAGACTTGGACTTATG	
@SRR1785046.15761233	-33	1000	-1033	GATTTTTCAGGAAGACTTGG	CGGGAAGACTTGGATTTTCG	
