# Supplementary material for: ciRS-7 exonic sequence is embedded in a long non-coding RNA locus
Source: PLoS Genet. 2017 Dec 13;13(12):e1007114. doi: 10.1371/journal.pgen.1007114 (PMC5745005; doi:10.1371/journal.pgen.1007114)
Supplement: S5 File — (PDF) [file pgen.1007114.s021.pdf]

```
import pandas as pd
import numpy as np

path = '/directory/containing/file/'
fn = 'correlation_values.csv'

# import file
correlations = pd.read_csv(path + fn, sep=',', index_col = 0)
correlations['gene'] = [i.split('_')[0] for i in correlations.index]

# subset the null and cdr1 loci
null = correlations[correlations['gene'] != 'linc-cdr1as']
cdr1 = correlations[correlations['gene'] == 'linc-cdr1as']

# set the threshold to .005, and identify the relevant position in the null data set
threshold = .005
threshold = int(np.floor(len(null) * threshold))

# make a Series to store the information
cutoffs = pd.Series(index = correlations.columns[:6])

# for each mark, sort the null values, and save the correlation value corresponding to the
threshold
for i in cutoffs.index:
    temp = null.sort_values(i, ascending=False)
    cutoffs[i] = temp.iloc[threshold][i]

# for each mark, print the information about the cdr1 locus
for i in cutoffs.index:
    min_correlation = cutoffs[i]
    temp = cdr1[cdr1[i] > min_correlation]
    print(i)
    print('the minimum correlation cutoff for ' + str(i) + ' is ' + str(min_correlation))
    print('there are ' + str(len(temp)) + ' marks for ' + str(i) + ' across the ASINC locus')
    print(temp[i], '\n')

##################################
# output below in hg19 coordinates (to convert to hg38 coordinates, add 917,835 to each value)
##################################
# H3K27ac
# the minimum correlation cutoff for H3K27ac is 0.755729648501
# there are 14 marks for H3K27ac across the ASINC locus
# name
# linc-cdr1as_82_chrX-139782918-139783417     0.925624
```

```
# linc-cdr1as_83_chrX-139783418-139783917     0.925624
# linc-cdr1as_108_chrX-139795918-139796417    0.967731
# linc-cdr1as_124_chrX-139803918-139804417    0.925624
# linc-cdr1as_125_chrX-139804418-139804917    0.925624
# linc-cdr1as_138_chrX-139810918-139811417    0.925624
# linc-cdr1as_139_chrX-139811418-139811917    0.925624
# linc-cdr1as_171_chrX-139827418-139827917    0.925624
# linc-cdr1as_189_chrX-139836418-139836917    0.925624
# linc-cdr1as_211_chrX-139847418-139847917    0.852785
# linc-cdr1as_213_chrX-139848418-139848917    0.925624
# linc-cdr1as_214_chrX-139848918-139849417    0.925624
# linc-cdr1as_215_chrX-139849418-139849917    0.925624
# linc-cdr1as_236_chrX-139859918-139860417    0.925624
# Name: H3K27ac, dtype: float64

# H3K27me3
# the minimum correlation cutoff for H3K27me3 is 0.510416628079
# there are 0 marks for H3K27me3 across the ASINC locus
# Series([], Name: H3K27me3, dtype: float64)

# H3K36me3
# the minimum correlation cutoff for H3K36me3 is 0.312885568512
# there are 23 marks for H3K36me3 across the ASINC locus
# name
# linc-cdr1as_55_chrX-139769418-139769917     0.925624
# linc-cdr1as_128_chrX-139805918-139806417    0.595254
# linc-cdr1as_131_chrX-139807418-139807917    0.845974
# linc-cdr1as_144_chrX-139813918-139814417    0.925624
# linc-cdr1as_153_chrX-139818418-139818917    0.925624
# linc-cdr1as_167_chrX-139825418-139825917    0.663930
# linc-cdr1as_180_chrX-139831918-139832417    0.925624
# linc-cdr1as_227_chrX-139855418-139855917    0.334574
# linc-cdr1as_228_chrX-139855918-139856417    0.562100
# linc-cdr1as_231_chrX-139857418-139857917    0.495208
# linc-cdr1as_245_chrX-139864418-139864917    0.444242
# linc-cdr1as_246_chrX-139864918-139865417    0.466550
# linc-cdr1as_247_chrX-139865418-139865917    0.409077
# linc-cdr1as_248_chrX-139865918-139866417    0.457302
# linc-cdr1as_250_chrX-139866918-139867417    0.925624
# linc-cdr1as_252_chrX-139867918-139868417    0.625719
# linc-cdr1as_254_chrX-139868918-139869417    0.925624
# linc-cdr1as_255_chrX-139869418-139869917    0.925624
# linc-cdr1as_261_chrX-139872418-139872917    0.925624
# linc-cdr1as_262_chrX-139872918-139873417    0.925624
```

```
# linc-cdr1as_277_chrX-139880418-139880917    0.757599
# linc-cdr1as_296_chrX-139889918-139890417    0.925624
# linc-cdr1as_304_chrX-139893918-139894417    0.925624
# Name: H3K36me3, dtype: float64

# H3K4me1
# the minimum correlation cutoff for H3K4me1 is 0.349323049155
# there are 6 marks for H3K4me1 across the ASINC locus
# name
# linc-cdr1as_83_chrX-139783418-139783917     0.925624
# linc-cdr1as_103_chrX-139793418-139793917    0.795332
# linc-cdr1as_112_chrX-139797918-139798417    0.619469
# linc-cdr1as_189_chrX-139836418-139836917    0.925624
# linc-cdr1as_205_chrX-139844418-139844917    0.400019
# linc-cdr1as_215_chrX-139849418-139849917    0.853667
# Name: H3K4me1, dtype: float64

# H3K4me3
# the minimum correlation cutoff for H3K4me3 is 0.445950743512
# there are 4 marks for H3K4me3 across the ASINC locus
# name
# linc-cdr1as_102_chrX-139792918-139793417    0.450851
# linc-cdr1as_107_chrX-139795418-139795917    0.745253
# linc-cdr1as_205_chrX-139844418-139844917    0.864989
# linc-cdr1as_216_chrX-139849918-139850417    0.549230
# Name: H3K4me3, dtype: float64

# H3K9me3
# the minimum correlation cutoff for H3K9me3 is 0.925624375777
# there are 0 marks for H3K9me3 across the ASINC locus
# Series([], Name: H3K9me3, dtype: float64)
```
